# Supplementary material for: Lysyl oxidase-like 3 restrains mitochondrial ferroptosis to promote liver cancer chemoresistance by stabilizing dihydroorotate dehydrogenase
Source: Nat Commun. 2023 May 30;14:3123. doi: 10.1038/s41467-023-38753-6 (PMC10229567; doi:10.1038/s41467-023-38753-6)
Supplement: Supplementary file 1 — Supplementary Information [file 41467_2023_38753_MOESM1_ESM.pdf]

Supplementary Information

Lysyl oxidase-like 3 restrains mitochondrial ferroptosis to promote liver cancer chemoresistance by stabilizing dihydroorotate dehydrogenase

Meixiao Zhan[1, #], Yufeng Ding[2, #, *], Shanzhou Huang[3, #], Yuhang Liu[2], Jing Xiao[1], Hua Yu [2, *], Ligong Lu[1, *], Xiongjun Wang[2, *]

[1]Zhuhai Interventional Medical Center, Guangdong Provincial Key Laboratory of Tumour Interventional Diagnosis and Treatment, Zhuhai People's Hospital, Zhuhai Hospital affiliated with Jinan University, Zhuhai, Guangdong, 519000, China

[2]Precise Genome Engineering Center, School of Life Sciences, Guangzhou University, Guangzhou 510006, China

[3]Department of General Surgery, Guangdong Provincial People's Hospital, Guangdong Academy of Medical Sciences, Guangzhou 510080, China.

[#]These authors contributed equally

[*]Correspondence: wangxiongjun@gzhu.edu.cn (X.W), luligong1969@jnu.edu.cn (L.L), yuhua@sibcb.ac.cn (H.Y), yfding@sibs.ac.cn (Y.D).

Supplementary Figure and Legends

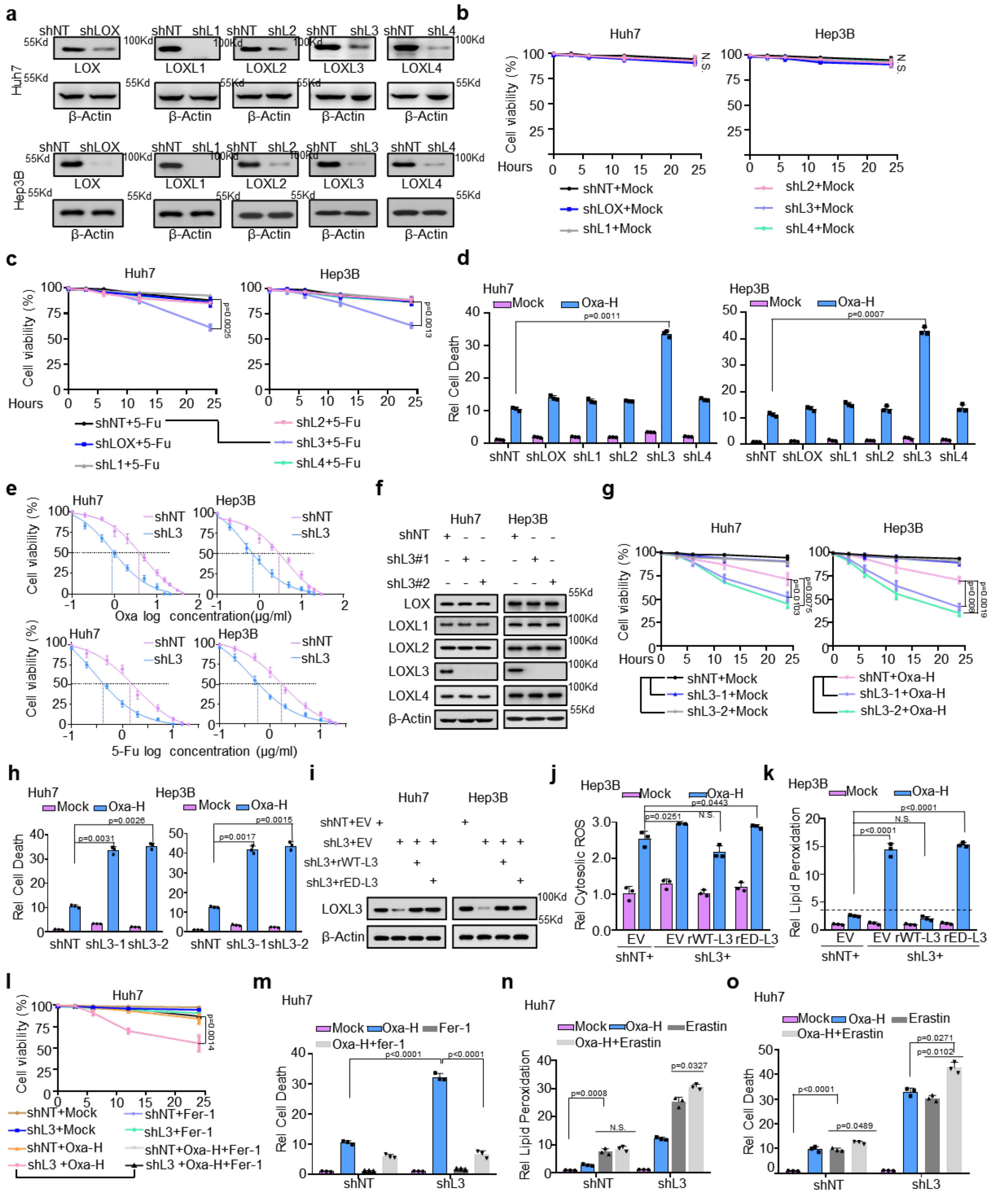

Supplementary Figure S1a-o Referred to Figure 1. a WB of Huh7 or Hep3B cells stably expressing shNT or shLOX, shL1, shL2, shL3, shL4. **b** Cell viability of Huh7 or Hep3B cells stably expressing shNT or shLOX, shL1, shL2, shL3, shL4. **c** Cell viability of Huh7 or Hep3B cells stably expressing shNT or shLOX, shL1, shL2, shL3, shL4 treated with 5-Fu in the indicated time points. **d** Huh7 or Hep3B cells stably expressing shNT or shLOX, shL1, shL2, shL3, shL4 were treated with or without Oxa-H for 24 hours and collected for cell death measurement. **e** IC50 values of Oxaliplatin or 5-Fu on shNT or shL3 of Huh7 or Hep3B cells determined by drug response curve. **f-h** WB of Huh7 or Hep3B cells stably expressing shNT or shL3-1, shL3-2 and other LOX family proteins expression level in indicated cells (**f**). The cell viability (**g**) and cell death (**h**) of indicated cells were measured under the treatment of DMSO or Oxa-H. **i** WB of restored expression of WT- or ED-LOXL3 in Huh7 or Hep3B shLOXL3 cells. ED, enzyme

dead, H607/609Q. **j-k** Cytosolic ROS levels of indicated Hep3B cells with restored expression of WT- or ED-LOXL3 under Oxa-H treatment were measured by fluorescence intensity at Ex/Em=495/529 nm (**j**) and respective lipid peroxidation levels were assessed by flow cytometry using BODIPY C11(**k**). **l-m** Huh7 cells stably expressing shNT or shL3 were treated with or without Oxa-H, ferrostatin-1(Fer-1) for the indicated time points, then cell viability (**l**) and cell death (**m**) were assessed. **n-o** Huh7 cells stably expressing shNT or shL3 were treated with or without Oxa-H, Erastin, or a combination of Oxa-H and Erastin. Oxa-H, high-dose Oxaliplatin, 5 μg ml^{-1}; 5-Fu, 2 μg ml^{-1}; Oxa-L, low-dose Oxaliplatin, 1 μg ml^{-1}; Erastin, 10 μM; Ferrostatin-1, 5 μM. For **b-e**, **g-h**, **j-o**, data represent means ± SEM of three independent experiments (n=3) and the statistical analysis was calculated by two-way ANOVA for multiple comparisons (**b-c**, **e**, **g**, **l**), one-way ANOVA with Tukey's HSD post hoc test (**d**, **h**, **j-k**, **m-o**). Source data are provided as a Source Data file.

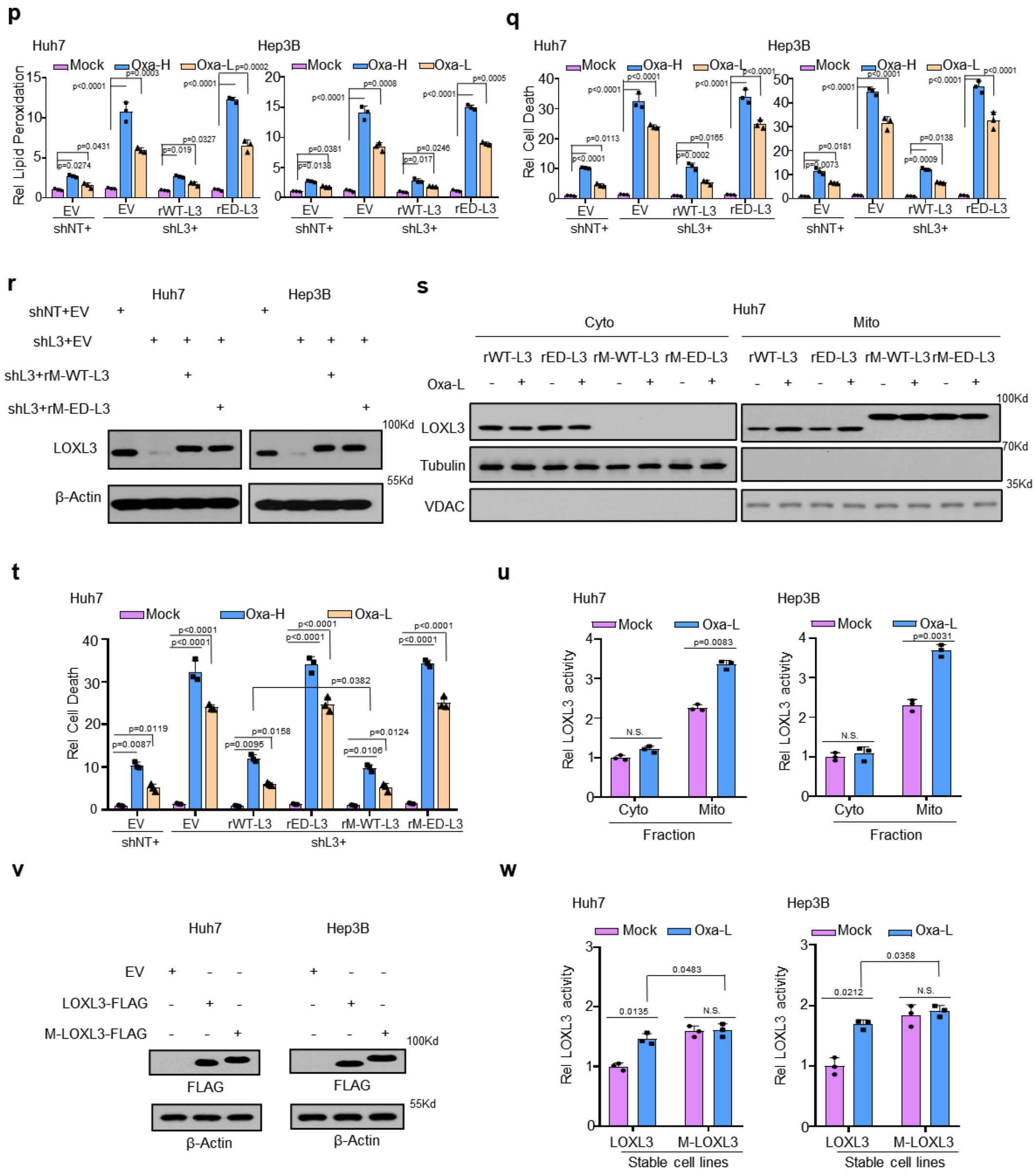

Supplementary Figure S1p-w Referred to Figure 1. p-q Huh7 or Hep3B stably expressing shNT with EV or expressing shL3 with EV, restored WT- or ED-LOXL3 were treated with or without Oxa-H/L. The treatment time for lipid peroxidation (**p**) was 6 hours while the treatment time for cell death (**q**) was 24 hours. **r-t** The successful construction of restored expression of mitochondrial peptide tagged WT- or ED-LOXL3 in Huh7 or Hep3B cells expressing shL3 were confirmed by WB (**r**). The mitochondrial localization of above cells was ascertained by fractionation assays (**s**), then cell death under Oxa-H/L (**t**) was assessed. **u** Huh7 or Hep3B cells stably expressing LOXL3-FLAG with or without N terminal tagged mitochondrial signal peptide were treated with or without Oxa-L for half hour, then LOXL3-FLAG were respectively enriched and purified from cytosol or mitochondrial of above cells, for activity determination. **v-w** WB was performed to confirm the construction of the indicated cells stably expressing LOXL3-FLAG with or without N terminal tagged mitochondrial signal peptide, using indicated antibodies (**v**). Then, the successful constructed cells were treated with or without Oxa-L for half hour, LOXL3-FLAG was measured for LOXL3 activity (**w**). Oxa-H, high-dose Oxaliplatin, 5 $\mu g\ ml^{-1}$; Oxa-L, low-dose Oxaliplatin, 1 $\mu g\ ml^{-1}$. For **p-q**, **t-u**, **w**, data represent means ± SEM of three independent experiments (n=3) and the statistical analysis was calculated by one-way ANOVA with Tukey's HSD post hoc test (**p-q**, **t-u**, **w**). Source data are provided as a Source Data file.

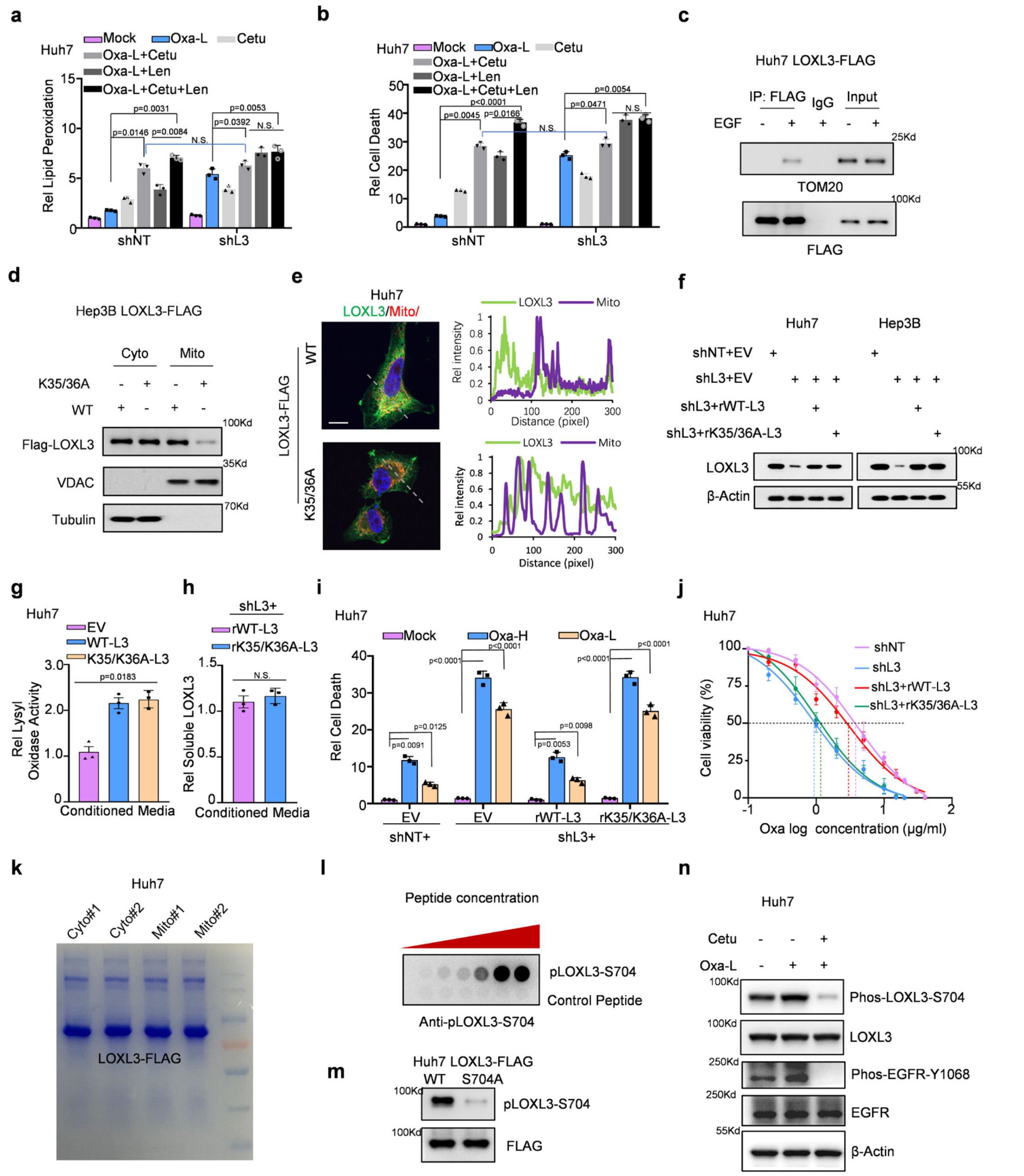

Supplementary Figure 2 Referred to Figure 2. a-b Huh7 cells stably expressing shNT or shLOXL3 were treated with Oxa-L, Cetu, Lenva or their combination for lipid peroxidation determination (**a**) and cell death level measurement (**b**). **c** Co-IP between LOXL3 and TOM20 was performed using Huh7 cells stably expressing LOXL3-FLAG. **d** Fractionation was performed using Hep3B cells stably expressing WT or K35/36A mutant LOXL3-FLAG. **e** Huh7 or Hep3B cells stably expressing WT or K35/36A mutant LOXL3-FLAG were fixed for IF. Scale bars: 20 μm. **f** WB confirmed the successful construction of restored expression of WT- or K35/36A-LOXL3 in Huh7 or Hep3B cells expressing shLOXL3. **g-h** The conditioned media (CM) was collected from the cells expressing EV, WT-L3 or K35/36A-L3 for lysyl oxidase activity measurement (**g**). CM of shL3 cells with restored expression of WT-L3 or K35/36A-L3 was collected for secreted LOXL3 measurement by ELISA (**h**). **i** Huh7 cells expressing shNT with EV or expressing shL3 with EV, WT- or K35/36A-mutant LOXL3 were treated with or without Oxa-H/L for 24 hours and collected for cell death measurement. **j** IC50 values of Oxaliplatin on the indicated groups of Huh7 cells

determined by drug response curve. **k** The fraction of cytosol and mitochondrial using Huh7 cells expressing LOXL3-FLAG was performed. Then, LOXL3-FLAG was purified, separated by SDS-PAGE and stained by Coomassie Brilliant Blue. **l** Dot blot using antibody against phosphorylated LOXL3 at S704. **m** WB of confirming the specificity of antibody against phosphorylated LOXL3 at S704. **n** WB of Huh7 cells treated with Oxa-L or Cetu for 12 hours, using indicated antibodies. Oxa-L, low-dose Oxaliplatin, 1 $\mu g\ ml^{-1}$; Cetu, Cetuximab, 20 $\mu g\ ml^{-1}$; Lenva, Lenvatinib, 2.5 μM. For **a**-**b**, **g**-**j**, data represent means ± SEM of three independent experiments (n=3) and the statistical analysis was calculated by one-way ANOVA with Tukey's HSD post hoc test (**a**-**b**, **i**), two-tailed Student's t test (**g**-**h**) and two-way ANOVA for multiple comparisons (**j**). Source data are provided as a Source Data file.

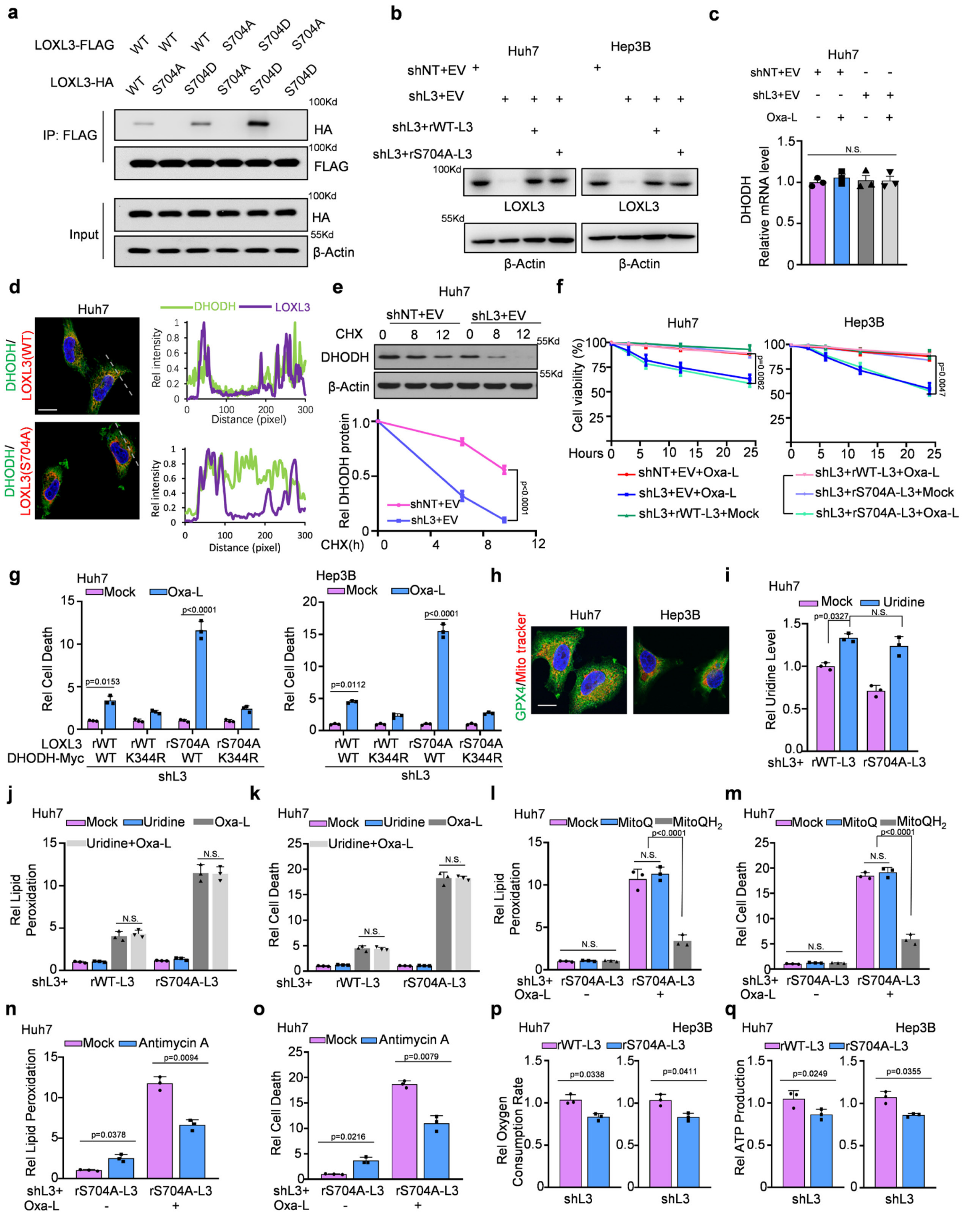

Supplementary Figure 3 Referred to Figure 3. a Immunoprecipitation (IP) of Huh7 cells transfected with C terminal FLAG or HA-tagged LOXL3 (WT or mutant) using FLAG, bloting with HA to test the homodimerization of LOXL3. **b** WB of Huh7 or Hep3B shLOXL3 cells reintroduced WT or S704A mutant LOXL3. **c** qRT-PCR of Huh7 shLOXL3 cells with or without restored expression of WT- or S704A-mutant LOXL3 were treated with or without Oxa-L. **d** IF of Huh7 cells expressing DHODH and LOXL3-FLAG (WT or S704A mutant) by indicated antibodies. Scale bars: 20 μm. **e** WB of Huh7 expressing shNT or shL3 cells treated with CHX (**e**, upper). The protein decay was analyzed (**e**, lower). **f** Cell viability of indicated Huh7 or Hep3B cells treated with or without Oxa-L. **g** Relative cell death of indicated Huh7 or Hep3B cells. **h** IF of Huh7 or Hep3B cells using antibody against GPX4 and MitoTracker marking mitochondrial. Scale bars: 20 μm. **i-k**

Intracellular uridine level of indicated Huh7 cells supplemented with or without uridine for 24 hours (**i**). Above cells were treated with or without Oxa-L and collected for lipid peroxidation (**j**) and cell death measurement (**k**). **l-m** Relative lipid peroxidation (**l**) and cell death level (**m**) of indicated Huh7 cells that treated with or without Oxa-L and combined with mitoQ or mitoQH$_2$ treatment. **n-o** Relative lipid peroxidation (**n**) and cell death level (**o**) of indicated Huh7 cells that treated with or without Oxa-L and combined with Antimycin A treatment. **p-q** Huh7 shLOXL3 cells that reintroduced with WT or S704A mutant LOXL3 were treated with or without Oxa-L to measure ATP production (**p**) and oxygen consumption rate level (**q**). CHX, cycloheximide, 2 μM. Oxa-L, low-dose Oxaliplatin, 1 μg ml^{-1}; uridine, 50 μM. mitoQ, 10 μM. mitoQH$_2$, 10 μM. Antimycin A, 10 μM. For **c**, **e-g**, **i-q**, data represent means ± SEM of three independent experiments (n=3) and the statistical analysis was calculated by two-way ANOVA for multiple comparisons (**e-f**), one-way ANOVA with Tukey's HSD post hoc test (**c**, **g**, **i-o**) and two-tailed Student's t test (**p-q**). Source data are provided as a Source Data file.

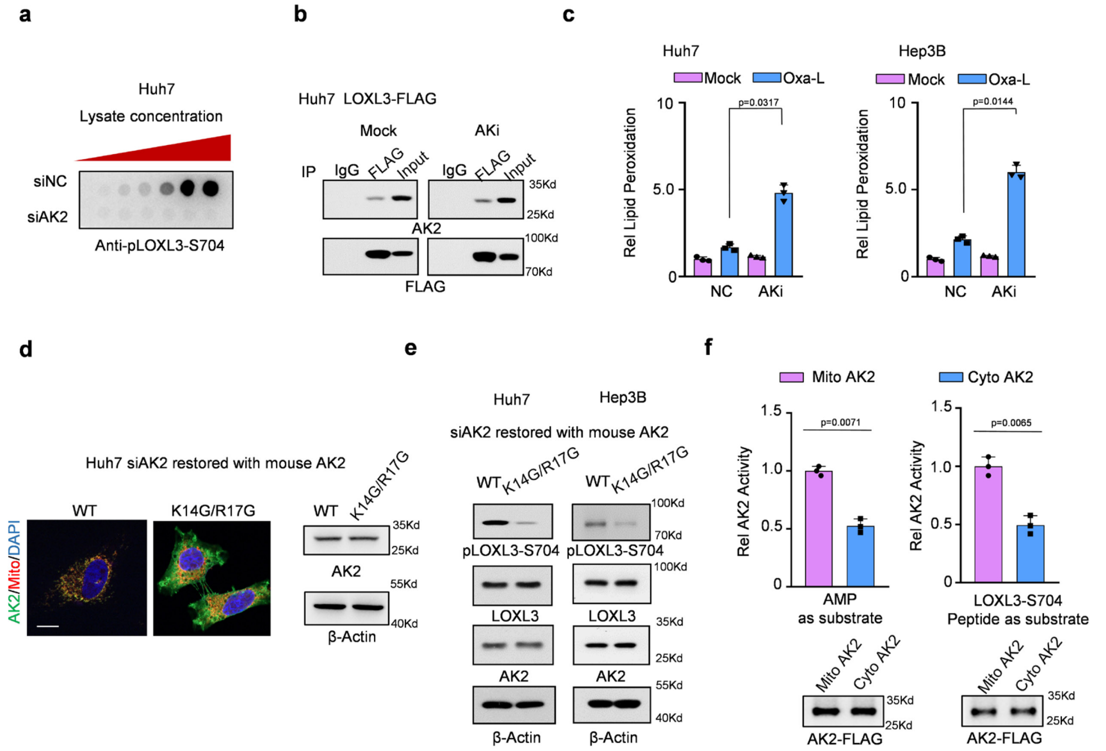

Supplementary Figure 4 Referred to Figure 4. a Huh7 cells were transfected with siRNA targeting human AK2 or negative control for 48rs and then collected to extract total proteins. Dot blot was performed using the indicated antibodies. **b** Huh7 cells stably expressing LOXL3-FLAG were treated with or without adenosine kinase inhibitor ABT-702 (1 μM) for 6 hours, and then collected for performing Co-IP using antibodies against FLAG. WB was performed using indicated antibodies. **c** Huh7 or Hep3B cells were treated with or without adenosine kinase inhibitor ABT-702 (AKi, 1μM) for 24 hours and collected for the lipid peroxidation determination. NC, negative control. **d** Huh7 cells transfected with siRNA targeting human AK2 were restored expression with mouse WT or K14G/R17G mutant AK2 for 48 hours and collected, fixed and stained with an antibody against AK2 and MitoTracker. Scale bars: 20 μm. **e** Huh7 or Hep3B cells with depletion of AK2 were restored with mouse WT or K14G/R17G mutant AK2 and indicated antibody against

pLOXL3-S704 was used for WB. **f** The activity measurement of purified fractional AK2-Flag using AMP (**f,** left) or LOXL3-S704 peptide as substrate (**f**, right). Oxa-L, low-dose Oxaliplatin, 1 μg ml^{-1}. For **c** and **f**, data were represented as means ± SEM of three independent experiments (n=3) and the statistical analysis was calculated by one-way ANOVA with Tukey's HSD post hoc test (**c**) and two-tailed Student's t test (**f**). Source data are provided as a Source Data file.

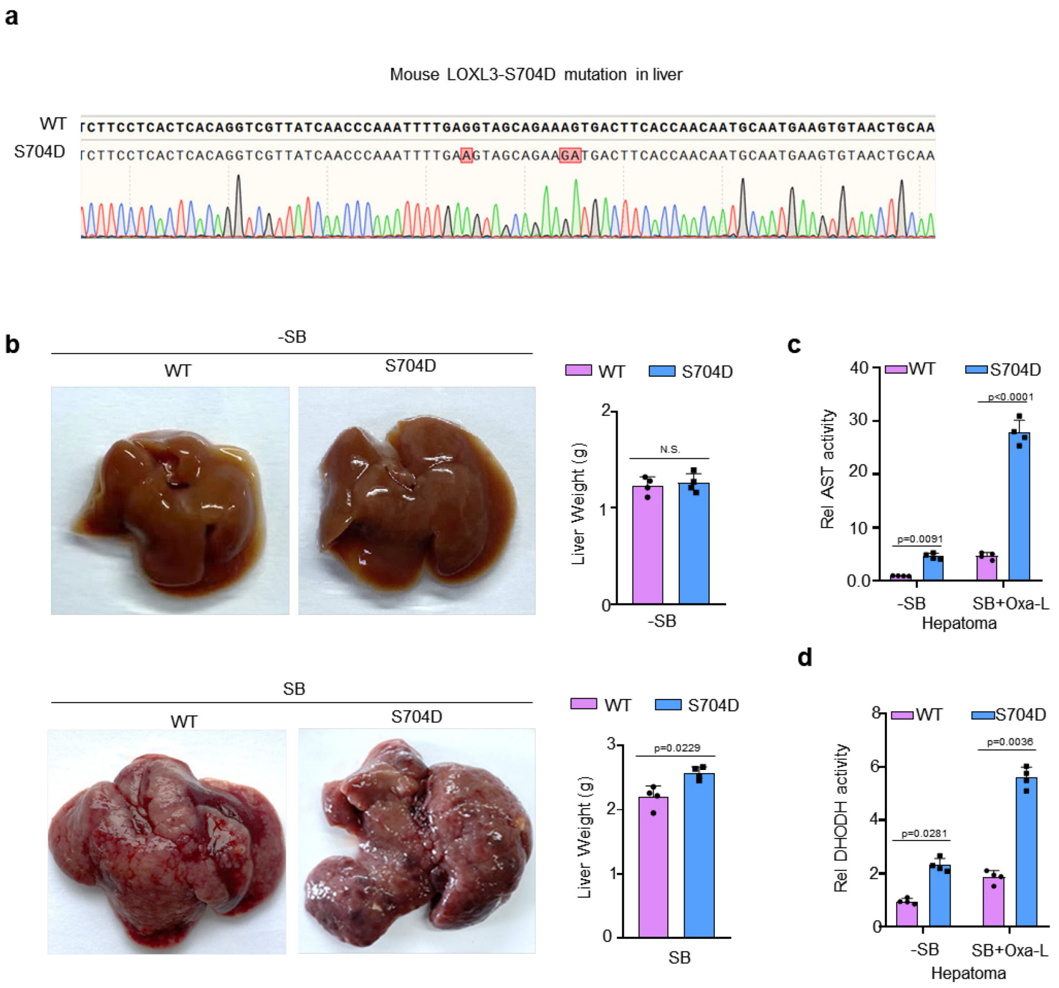

Supplementary Figure 5 Referred to Figure 5. **a** The DNA genomic was extracted from mouse liver and sequencing was performed to ascertain the mutation of S704 to D704. **b** After 6 weeks induction with SB mouse model or not, the representative livers of WT and *Loxl3-S704D* mutant mice are displayed, and liver weight was measured. n=4 in each group. **c-d** ALT activity (**c**) and DHODH activity determination (**d**) in livers which were dissected from mice induced with or without of SB transposon and a consequent treatment of with Oxa-L. n=4 in each group. SB, sleeping beauty transposon; Oxa-L, low-dose Oxaliplatin, 1 mg kg^{-1} weight, oral gavage in animal treatment. For **b-d**, data represent means ± SEM of individual mice group (n=4 in each group) and the statistical analysis was calculated by two-tailed Student's t test (**b-d**). Source data are provided as a Source Data file.

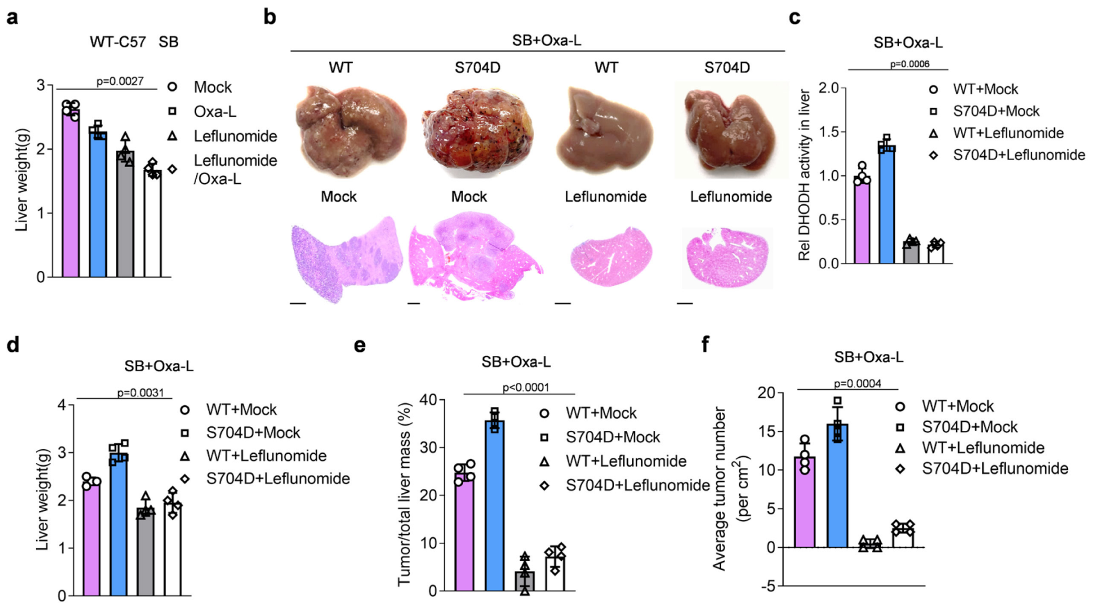

Supplementary Figure S6 Referred to Figure 6. a Livers were dissected from the experimental groups in Figure 6g, then the liver weight was measured. **b-f** SB induced liver tumors were preliminarily achieved within 8 weeks for *S704D-Loxl3* or WT mice (n=4 in each group). Low-dose Oxaliplatin was administered in 12 days with once every 4 days for pre-administration. After that, low-dose Oxaliplatin (1 mg kg^{-1} weight) plus with vehicle or low-dose Oxaliplatin (1 mg kg^{-1} weight) plus with DHODH inhibitor Leflunomide (0.2 mg kg^{-1} weight) was treated intraperitoneally 3 times a week for 12 weeks, then the livers were dissected (**b**, upper). HE staining was performed using the upper representative livers (**b**, lower). DHODH activity in liver tumor was tested (**c**). The liver weight (**d**), The ratio of tumor in liver mass (**e**) and the average tumor number (**f**) were analyzed and calculated. Scale bars: 2 mm (**b**, lower). Oxa-L, low-dose Oxaliplatin,1 mg kg^{-1} weight, intraperitoneal injection in animal treatment; Leflunomide (0.2 mg kg^{-1} weight, intraperitoneal injection in animal treatment. For **a**, **c-f**, data represent means ± SEM of individual mice group (n=4 in each group), and the statistical analysis was calculated by one-way ANOVA with Tukey's HSD post hoc test (**a**, **c-f**). Source data are provided as a Source Data file.

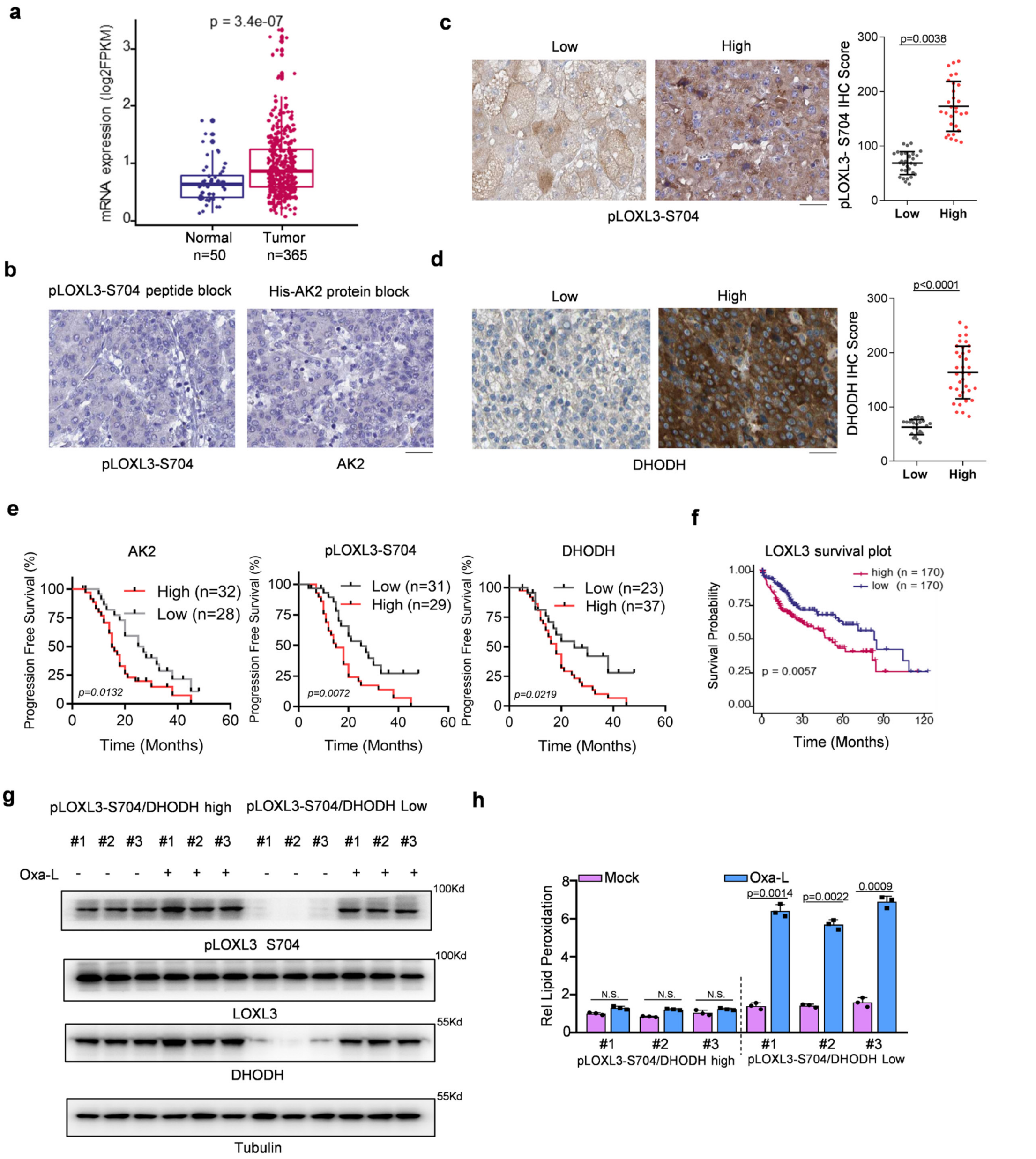

Supplementary Figure S7 Referred to Figure 7. a LOXL3 mRNA expression levels in normal (n=50) and tumor (n=365) tissues in the TCGA-LIHC cohort. The FPKM values were log2 transformed. Boxes represent data within the 25th to 75th percentiles. Whiskers depict the range of all data points. Horizontal lines within boxes represent mean values. **b** The antibody specificity in tissue level was tested by incubating peptide or protein to block the antigen. Scale bars: 200 µm. **c-d** IHC of HCC clinical samples using antibodies against pLOXL3-S704 (**c**) or DHODH (**d**). The patients were divided into two groups, high and low, according to histology quantification. Representative images are shown. Scale bars: 200 µm. **e** Kaplan-Meier plot analysis of progression free survival of the HCC patients, respectively based on their AK/LOXL3/DHODH histological expression. **f** Overall survival of patients with different LOXL3 expression levels in the TCGA-LIHC cohort. Patients were divided into high and low groups by the median of LOXL3 mRNA expression levels. **g-h** The levels of pLOXL3-S704 and DHODH in PDXs samples were confirmed by WB (**g**) while the lipid peroxidation determination was performed and calculated using flow

cytometry(**h**). For **a**, **c-d**, **h**, data represent means ± SEM of individual human HCC sample groups (**a**, **c-d**) or individual PDX groups (**h**). The statistical analysis was calculated by two tailed Mann-Whitney U-test (**a**, **c-d**), the log rank test (**e-f**), one-way ANOVA with Tukey's HSD post hoc test (**h**). Source data are provided as a Source Data file.

Supplementary Table 1.

The clinicopathological information of human HCC patients	
**Clinicopathological parameters**	**No. of specimens**
Sex	
Male, n (%)	49 (81.7)
Female, n (%)	11 (18.3)
Age	
≤ 50-year-old, n (%)	16 (26.7)
> 50-year-old, n (%)	44 (73.3)
Antigen (CEA)	
≤ 5 ng/mL, n (%)	51 (85)
> 5 ng/mL, n (%)	9 (15)
α-fetoprotein (AFP)	
≤ 20 ng/mL, n (%)	28 (46.7)
> 20 ng/mL, n (%)	32 (53.3)
Cirrhosis	
Present, n (%)	41 (68.3)
Absent, n (%)	19 (31.7)
Tumor size, median (range)	
≤ 5 cm, n (%)	34 (56.7)
> 5 cm, n (%)	26 (43.3)
Multinodular	
Yes, n (%)	11 (18.3)
No, n (%)	49 (81.7)
Tumor pathological grade	
I	18 (30)
II	26 (43.3)
III	16 (26.7)
Vascular invasion	
Present	18 (30)
Absent	42 (70)
HBV	
Positive	39 (0.65)
Negative	21 (0.35)
Liver invasion	
Present, n (%)	27 (0.45)

Absent, n (%)	33 (0.55)
**Tumor microsatellite formation**	
Present, n (%)	43 (71.7)
Absent, n (%)	17 (28.3)
**Cellular differentiation**	
Edmondson grade I-II, n (%)	36 (60)
Edmondson grade I-II, n (%)	24 (40)
**TNM classification**	
I-II, n (%)	33 (0.55)
III-IV, n (%)	27 (0.45)

Supplementary Table 2.

The clinicopathological information of human HCC patients for PDX										
PDX number	Anatomical site	Diagnosis	Biospecimen type	Grade	Stage	Multinodular	Vascular invasion	HBV	pLOXL3-S704 (%)	DHODH (%)
PDX1	Liver	HCC	tumor tissue	II-III	T2N1M0	Absent	Absent	Absent	93	85
PDX2	Liver	HCC	tumor tissue	II-III	T2N1M0	Present	Absent	Absent	83	76
PDX3	Liver	HCC	tumor tissue	II-III	T2N0M0	Absent	Absent	Absent	90	65
PDX4	Liver	HCC	tumor tissue	II-III	T2N1M0	Absent	Absent	Present	26	34
PDX5	Liver	HCC	tumor tissue	II-III	T2N0M0	Absent	Absent	Absent	13	22
PDX6	Liver	HCC	tumor tissue	II-III	T2N0M0	Absent	Present	Absent	11	29

Supplementary Table 3.

**shRNA or siRNA sequences and RT-qPCR primer sequences.**		
shRNA or siRNA sequences		
Target	shRNA/siRNA name	Target sequence
LOX	shLOX	AGACTGCCAGTGGATTGATAT
LOXL1	shL1	ACGTGGTGAGATGCAACATTC
LOXL2	shL2	GGCAATGAGAAGTCCATTATA
LOXL3	shL3	GGACCCACAGTGCCAAATATG
LOXL4	shL4	TTGGACCAGTGCGGGTCTAAT
LOXL3	shL3-1	ACTGGGACTCTGGGAATATAA
LOXL3	shL3-2	CATCTTCACTCACTATGATAT
AK2	siAK2-1	GAAACTGGTGAGTGATGAAtt
AK2	siAK2-2	CGATGTCGTGTTCGCAAGCtt
RT-qPCR primer sequences		
Target	Forward primer	Reverse primer
DHODH	GTTCTGGGCCATAAATTCCGA	TCTGGGTCTAGGGTTTCCTTC
β-Actin	CACCATTGGCAATGAGCGGTTC	AGGTCTTTGCGGATGTCCACGT
