# Supplementary material for: Lysyl oxidase-like 3 restrains mitochondrial ferroptosis to promote liver cancer chemoresistance by stabilizing dihydroorotate dehydrogenase
Source: Nat Commun. 2023 May 30;14:3123. doi: 10.1038/s41467-023-38753-6 (PMC10229567; doi:10.1038/s41467-023-38753-6)
Supplement: Supplementary file 3 — Reporting Summary [file 41467_2023_38753_MOESM3_ESM.pdf]

Corresponding author(s): Xiongjun Wang

Last updated by author(s): May 1, 2023

Reporting Summary

Nature Portfolio wishes to improve the reproducibility of the work that we publish. This form provides structure for consistency and transparency in reporting. For further information on Nature Portfolio policies, see our Editorial Policies and the Editorial Policy Checklist.

Statistics

For all statistical analyses, confirm that the following items are present in the figure legend, table legend, main text, or Methods section.

n/a	Confirmed	
☐	☒	The exact sample size (n) for each experimental group/condition, given as a discrete number and unit of measurement
☐	☒	A statement on whether measurements were taken from distinct samples or whether the same sample was measured repeatedly
☐	☒	The statistical test(s) used AND whether they are one- or two-sided<br>*Only common tests should be described solely by name; describe more complex techniques in the Methods section.*
☒	☐	A description of all covariates tested
☒	☐	A description of any assumptions or corrections, such as tests of normality and adjustment for multiple comparisons
☐	☒	A full description of the statistical parameters including central tendency (e.g. means) or other basic estimates (e.g. regression coefficient) AND variation (e.g. standard deviation) or associated estimates of uncertainty (e.g. confidence intervals)
☐	☒	For null hypothesis testing, the test statistic (e.g. F, t, r) with confidence intervals, effect sizes, degrees of freedom and P value noted<br>*Give P values as exact values whenever suitable.*
☒	☐	For Bayesian analysis, information on the choice of priors and Markov chain Monte Carlo settings
☒	☐	For hierarchical and complex designs, identification of the appropriate level for tests and full reporting of outcomes
☐	☒	Estimates of effect sizes (e.g. Cohen's d, Pearson's r), indicating how they were calculated

Our web collection on statistics for biologists contains articles on many of the points above.

Software and code

Policy information about availability of computer code

Data collection	Imaging: Zeiss Axio Imager 2 microscope and Zeiss LSM 880 confocal platform (Carl Zeiss)<br>Protein activity and cellular ROS measurement: Multi-Mode BioTek Synergy Neo Plate Reader (BioTek)<br>Protein modification data: Q Exactive HF mass spectrometer with HPLC Easy-nLC 1000 system (Thermo Fisher Scientific)<br>Lipid peroxidation data: BD FACSLyric<br>Ultrastructural data: Zeiss Libra 120 Transmission Electron Microscopy (Carl Zeiss)<br>RT-qPCR: QuantStudio 7 Flex Real-Time PCR System
Data analysis	The plate reader raw data were analyzed by VWorks software and BioTek Gen5 Reader Diagnostic v14.0. The MS raw data were analyzed using Proteome Discoverer 2.3 against the human Swiss-Prot database containing 20,231 sequences (downloaded in December 2017). Phosphopeptide matches were analyzed using MaxQuant 1.5.2.8, implemented in Proteome Discoverer, and were manually curated. The imaging raw data were obtained by ZEN software offered by Carl Zeiss, next analyzed by Image J. The flow cytometry raw data of lipid peroxidation data by BD FACSLyric were exported by BD FACSDiva™ Softwarea and furthyer analyzed by FlowJo v10.7. The Software GraphPad Prism software v9.0 was used to generate graphs with statistical analysis that was included in methods section.

For manuscripts utilizing custom algorithms or software that are central to the research but not yet described in published literature, software must be made available to editors and reviewers. We strongly encourage code deposition in a community repository (e.g. GitHub). See the Nature Portfolio guidelines for submitting code & software for further information.

Data

Policy information about availability of data

All manuscripts must include a data availability statement. This statement should provide the following information, where applicable:

- Accession codes, unique identifiers, or web links for publicly available datasets
- A description of any restrictions on data availability
- For clinical datasets or third party data, please ensure that the statement adheres to our policy

Source data for this study are provided with this paper. All relevant data in this study are available within the article, Supplementary information, Supplementary Data, or Source Data.

Human research participants

Policy information about studies involving human research participants and Sex and Gender in Research.

Reporting on sex and gender	This study did not apply to only one gender. Gender was not considered in the study design. The tumors from the participants were divided according to the blindly histological scoring of AK2/pLOXL3-S704/DHODH staining, for evaluation the clinical relevance of the regulation axis. Overall 49 male and 11 female patients were collected in our patient participants.
Population characteristics	Clinical summary of tumor tissue specimens from HCC patients for histological evaluation was listed in Supplementary Table 1. Clinical summary of tumor tissue specimens from HCC patients for PDX experiment was listed in Supplementary Table 2.
Recruitment	The study included patients who underwent surgical treatment, following with the FOLFOX (Oxaliplatin plus fluorouracil and leucovorin) chemotherapy regimen.
Ethics oversight	The use of pathological specimens, as well as the review of all pertinent patient records, was approved by the ethical standards of the institutional research committee and with the 1964 Declaration of Helsinki and its later amendments or comparable ethical standards. All patient samples were collected by the Department of Pathology with approval from the Research Ethics Committee of Zhuhai People's Hospital, and informed consent was obtained from the patients.

Note that full information on the approval of the study protocol must also be provided in the manuscript.

Field-specific reporting

Please select the one below that is the best fit for your research. If you are not sure, read the appropriate sections before making your selection.

☒ Life sciences ☐ Behavioural & social sciences ☐ Ecological, evolutionary & environmental sciences

For a reference copy of the document with all sections, see nature.com/documents/nr-reporting-summary-flat.pdf

Life sciences study design

All studies must disclose on these points even when the disclosure is negative.

Sample size	Experimental sample sizes were determined based on number of available, statistical methods, and previous reported data with related mouse models and extensive prior experience of identical or similar cell biology or biochemical assays or prior experiments using the GEMMs and xenograft models (see for example DOI: https://doi.org/10.1038/s41586-019-1340-y,or https://doi.org/10.1002/hep.31870). There are at least 4-6 mice in pairs in all animal models. The sample size is sufficient for statistical analysis in the research project. All representative images with consistent results from at least three biological replicates are shown to show statistical significance.
Data exclusions	None of the were data excluded.
Replication	Unless otherwise specified, all experiments with permissible conditions were repeated at least three independent times with similar results.
Randomization	All samples were allocated in random, including mice and cell line groups.
Blinding	Except the histology quantification and scoring, the investigators were not blinded to allocation experiments and outcome assessment

Reporting for specific materials, systems and methods

We require information from authors about some types of materials, experimental systems and methods used in many studies. Here, indicate whether each material, system or method listed is relevant to your study. If you are not sure if a list item applies to your research, read the appropriate section before selecting a response.

Materials & experimental systems

n/a	Involved in the study
☐	☒ Antibodies
☐	☒ Eukaryotic cell lines
☒	☐ Palaeontology and archaeology
☐	☒ Animals and other organisms
☒	☐ Clinical data
☒	☐ Dual use research of concern

Methods

n/a	Involved in the study
☒	☐ ChIP-seq
☐	☒ Flow cytometry
☒	☐ MRI-based neuroimaging

Antibodies

Antibodies used

Immunoprecipitation
Anti-Flag, Merck, clone: M2, #F1804
Western
Anti-β-Actin, Merck, clone:AC-74, Cat #a5316, 1:5000
Anti-Tubulin, Merck, Cat #ab3201, 1:5000
Anti-LaminB, Thermo Fisher Scientific, Cat #PA5-19468,1:1000
Anti-LOX, Thermo Fisher Scientific, Cat #PA1-16955, 1:1000
Anti-LOXL1, Thermo Fisher Scientific, Cat #MA5-41635, 1:1000
Anti-LOXL2, Thermo Fisher Scientific, Cat #711729, 1:1000
Anti-LOXL3, Thermo Fisher Scientific, Cat #PA5-48462, 1:1000
Anti-LOXL4, Thermo Fisher Scientific, Cat #PA5-115520, 1:1000
Anti-ACSL4, Thermo Fisher Scientific, Cat #PA5-30026, 1:1000
Anti-GPX4, Thermo Fisher Scientific, Cat #PA5-102521, 1:1000
Anti-DHODH, Thermo Fisher Scientific, Cat #PA5-89141, 1:1000
Anti-EGFR, Cell signaling Technology, Cat #4267, 1:1000
Anti-Phos-EGFR-Y1068, Cell signaling Technology, Cat #3777, 1:1000
Anti-Caspase 3, Thermo Fisher Scientific, clone: 9H19L2, Cat #700182, 1:1000
Anti-Cleaved Caspase-3, Cell signaling Technology, Cat #9661, 1:1000
Anti-TOM20, Thermo Fisher Scientific, Cat #MA5-34964, 1:1000
Anti-VDAC1, Thermo Fisher Scientific, Cat #PA1-954A, 1:1000
Anti-AK2, Thermo Fisher Scientific, Cat #11014-1-AP1:1000
Anti-phosphorylated-S704-LOXL3, generated by the investigators of the study, 1:500
IHC or IF
Anti-DHODH, Thermo Fisher Scientific, Cat #PA5-89141, 1:200
Anti-AK2, Thermo Fisher Scientific, Cat #11014-1-AP, 1:200
Anti-Ki67, Invitrogen, Cat #MA5-14520, 1:200
Anti-phosphorylated-S704-LOXL3, generated by the investigators of the study, 1:50

Validation

Primary antibodies used in this study are all commercial and validated by the manufactures for their respective application, except the phos-LOXL3-S704 antibody made by the investigators of the study.
Anti-Flag, Cat #F1804, was validated on the manufacture's website: https://www.sigmaaldrich.cn/CN/zh/product/sigma/f1804 and the amplification reference: https://doi.org/10.1038/ncomms7253
Anti-β-Actin, Cat #a5316, was validated on the manufacture's website: https://www.sigmaaldrich.cn/CN/zh/product/sigma/a5316
Anti-Tubulin, Cat #ab3201, was validated on the manufacture's website: https://www.sigmaaldrich.cn/CN/zh/product/mm/ab3201
Anti-LOX, Cat #PA1-16955, was validated on the manufacture's website: https://www.thermofisher.cn/cn/zh/antibody/product/LOX-Antibody-Polyclonal/PA1-16955
Anti-LOXL1, Cat #MA5-41635, was validated on the manufacture's website: https://www.thermofisher.cn/cn/zh/antibody/product/LOXL1-Antibody-clone-C1-Monoclonal/MA5-41635
Anti-LOXL2, Cat #711729, was validated on the manufacture's website: https://www.thermofisher.cn/cn/zh/antibody/product/LOXL2-Antibody-clone-7HCLC-Recombinant-Polyclonal/711729
Anti-LOXL3, Cat #PA5-48462, was validated on the manufacture's website: https://www.thermofisher.cn/cn/zh/antibody/product/LOXL3-Antibody-Polyclonal/PA5-48462
Anti-LOXL4, Cat #PA5-115520, was validated on the manufacture's website: https://www.thermofisher.cn/cn/zh/antibody/product/LOXL4-Antibody-Polyclonal/PA5-115520
Anti-ACSL4, Cat # PA5-30026, was validated on the manufacture's website: https://www.thermofisher.cn/cn/zh/antibody/product/ACSL4-Antibody-Polyclonal/PA5-30026
Anti-GPX4, Cat #PA5-102521), was validated on the manufacture's website: https://www.thermofisher.cn/cn/zh/antibody/product/GPX4-Antibody-Polyclonal/PA5-102521
Anti-DHODH, Cat #PA5-89141), was validated on the manufacture's website: https://www.thermofisher.cn/cn/zh/antibody/product/DHODH-Antibody-Polyclonal/PA5-89141
Anti-EGFR, Cat #4267, was validated on the manufacture's website: https://www.cellsignal.cn/products/primary-antibodies/egf-receptor-d38b1-xp-rabbit-mab/4267
Anti-Phos-EGFR-Y1068, Cat #, was validated on the manufacture's website: https://www.cellsignal.cn/products/primary-antibodies/phospho-egf-receptor-tyr1068-d7a5-xp-rabbit-mab/3777
Anti-Caspase 3, Cat #700182, was validated on the manufacture's website: https://www.thermofisher.cn/cn/zh/antibody/product/Caspase-3-Antibody-clone-9H19L2-Recombinant-Monoclonal/700182
Anti-Cleaved Caspase-3, Cat #9661, was validated on the manufacture's website: https://www.cellsignal.cn/products/primary-antibodies/cleaved-caspase-3-asp175-antibody/9661
Anti-TOM20, Cat #MA5-34964, was validated on the manufacture's website: https://www.thermofisher.cn/cn/zh/antibody/product/

TOM20-Antibody-Recombinant-Monoclonal/MA5-34964
Anti-VDAC1, Cat #PA1-954A, was validated on the manufacture's website: https://www.thermofisher.cn/cn/zh/antibody/product/VDAC-Antibody-Polyclonal/PA1-954A
Anti-AK2, Cat #11014-1-AP, was validated on the manufacture's website: https://www.thermofisher.cn/cn/zh/antibody/product/AK2-Antibody-Polyclonal/11014-1-AP
Anti-Ki67, Cat #MA5-14520, was validated on the manufacture's website: https://www.thermofisher.cn/cn/zh/antibody/product/Ki-67-Antibody-clone-SP6-Recombinant-Monoclonal/MA5-14520
Anti-Phos-LOXL3-S704, was generated by the investigators of this study, which was validated using dot blot and western blot in Supplemental Figure S2H-I.

Eukaryotic cell lines

Policy information about cell lines and Sex and Gender in Research

Cell line source(s)	Hep3B cell line: ATCC Cat#HB-8064<br>Huh7 cell line: Shanghai Institute of Biochemistry and Cell Biology (SIBCB), Chinese Academy of Sciences Cat# SCSP-526<br>293T ATCC Cat#CRL-3216
Authentication	All cell lines were authenticated by observing the morphology.
Mycoplasma contamination	There is no mycoplasma contamination in all cell lines.
Commonly misidentified lines<br>(See ICLAC register)	No commonly misidentified cell lines were used.

Animals and other research organisms

Policy information about studies involving animals; ARRIVE guidelines recommended for reporting animal research, and Sex and Gender in Research

Laboratory animals	For the xenograft study, 2 million Huh7-derived stable cells were subcutaneously injected into the left groins of randomized 6-week-old female athymic nude mice. Approximately three weeks after inoculation when the tumor size reached 50 mm2, low dosage of Oxaliplatin (1 mg/kg weight) administered intraperitoneally 3 times a week for 14 days. Nude mice were purchase from GemPharmatech Co., Ltd, China.<br>For PDX transplantation, frozen PDX tumors collected from the tumors of human HCC patients were prepared in a volume of 5-10 mm3, and then subcutaneously implanted into the flanks of 6-week-old NSG female mice following standard procedures. NSG mice were purchase from GemPharmatech Co., Ltd, China.<br>For Loxl3-S704D mutant mouse generation, Cas9 mRNA and sgRNA were generated by in vitro transcription and mutation donor ssDNA mixture were injected into the cytoplasm of fertilized eggs transferred into oviducts of pseudo-pregnant female mice at 0.5 dpc for the birth of mutated mice. The Loxl3-S704D mouse line was generated on a mixed FVB/N and C57BL/6 background. FVB/N and C57BL/6 mice were purchase from GemPharmatech Co., Ltd, China.<br>The mice scarified in the in vivo administration of Oxaliplatin (low-dose: 1 mg/kg weight) or in combination with Leflunomide to treat SB-driven hepatocarcinogenesis were C57BL/6 WT or Loxl3-S704D mutant mice. C57BL/6 WT mice were purchased from GemPharmatech Co., Ltd, China.
Wild animals	The study did not involve wild animals.
Reporting on sex	The investigator selected male mice for HTVI-induced HCC model maybe more efficiently established in male mice, according to our experience. In xenograft studies, we chose female mice, for the convenience of the experiment procedure and balance to the use of male mice in HTVI-induced HCC model, keeping the no gender bias in the in vivo experiments.
Field-collected samples	The study did not involve sample collected from the filed.
Ethics oversight	All mice received humane care, and all experimental procedures were performed in compliance with the Guide for the Care and Use of Laboratory Animals and approved by the animal care and use committee of Guangzhou University.

Note that full information on the approval of the study protocol must also be provided in the manuscript.

Flow Cytometry

Plots

Confirm that:

☒ The axis labels state the marker and fluorochrome used (e.g. CD4-FITC).

☒ The axis scales are clearly visible. Include numbers along axes only for bottom left plot of group (a 'group' is an analysis of identical markers).

☒ All plots are contour plots with outliers or pseudocolor plots.

☒ A numerical value for number of cells or percentage (with statistics) is provided.

Methodology

Sample preparation	For lipid Peroxidation determination, cells were seeded in DMEM, treated with Oxaliplatin for the indicated time. Then washed twice in HBSS and incubated in DMEM containing 2 mM BODIPY 581/591 C11 for 30 minutes at 37?. After that, cells were washed by PBS twice and digested with by using 0.25% Trypsin-EDTA. Cell suspension were permeabilized using Fixation/ Permeabilization Buffer for 1 hour at room temperature, protected from light. Further examination was performed through a BD laser analyser using PE-Texas Red (PE-TR) filter (measuring non-oxidized BODIPY-C11) and fluorescein isothiocyanate (FITC) (measuring oxidized BODIPY-C11).
Instrument	Data collection was performed on a BD FACSLyric flow cytometer.
Software	Analyses were performed by BD FACS Diva v8.0 and Flowjo.
Cell population abundance	At least 10,000 cells were captured and analyzed for each group.
Gating strategy	For all expreiments, cells were gated by FSC/SSC to exclude debris, followed by gating FSC-A and FSC-H to eliminate nonsinglets. Then target cell population for further analysis were gated by intensity.

☒ Tick this box to confirm that a figure exemplifying the gating strategy is provided in the Supplementary Information.
